# Phenolic Compounds and Antioxidant Activity of Bulb Extracts of Six *Lilium* Species Native to China

**DOI:** 10.3390/molecules17089361

**Published:** 2012-08-03

**Authors:** Lei Jin, Yanlong Zhang, Linmao Yan, Yulong Guo, Lixin Niu

**Affiliations:** 1 College of Horticulture, Northwest A&F University, Yangling, Shaanxi 712100, China; E-Mail: 1jinlei1@163.com; 2 College of Forestry, Northwest A&F University, Yangling, Shaanxi 712100, China; E-Mails: yanlinmao123@163.com (L.Y.); guoyulong1415121@163.com (Y.G.)

**Keywords:** *Lilium*, bulb, phenolic compounds, antioxidant activity

## Abstract

Lily (*Lilium*) is used as an important edible and medical plant species with a vague taxonomic classification and a long history in China. Bulbs of six *Lilium* species (*L. regale*, *L. concolor*, *L. pumilum*, *L. leucanthum*, *L. davidii var. unicolor* and *L. lancifolium*) native to China were investigated with a view to their exploitation as a potential source of natural antioxidants due to their phenolic composition and dietary antioxidant potential. The results showed that all bulb extracts exhibited strong antioxidant activities, which generally correlated positively with the total phenolic contents (r = 0.68 to 0.94), total flavonoid contents (r = 0.51 to 0.89) and total flavanol contents (r = 0.54 to 0.95). High-performance liquid chromatography (HPLC) analysis revealed that rutin and kaempferol were the major phenolic components in the extracts. Hierarchical cluster analysis showed that *L. regale* belonged to the group with high phenolic content and strong antioxidant power. *L. concolor* and *L. pumilum* were arranged in one group characterized by moderate phenolic content and antioxidant capacity, while *L. leucanthum*, *L. davidii var. unicolor* and *L. lancifolium* were clustered in the third group with low phenolic content and weak antioxidant activity. These strongly suggest that lily bulbs may serve as a potential source of natural antioxidant for food and pharmaceutical applications.

## 1. Introduction

The adverse effects of oxidative stress on human health have become a serious issue. Oxidative stress has been implicated in over one hundred human disease conditions, such as cancer, cardiovascular disease, aging and neurodegenerative diseases [1]. Reactive oxygen species (ROS), including free radicals and nonradical species, are the main factor causing oxidative stress [2]. Under pathological conditions or environmental stress, ROS levels can increase dramatically and a biological system’s ability to readily detoxify the reactive intermediates or to repair the resulting damage is interrupted. Consequently, the innate defense in the human body may not be enough for severe oxidative stress [3]. Hence, certain amounts of exogenous antioxidants are constantly required to maintain an adequate level of antioxidants in order to balance the ROS. 

Antioxidants are a class of substances that can delay or prevent the oxidation of oxidizable substrates. They exert their effects by scavenging ROS or preventing its production [4]. Recent epidemiological studies have revealed that synthetic phenolic antioxidants such as buthylated hydroxytoluene (BHT), buthylated hydroxyanisole (BHA) and *tert*-butylhydroquinone (TBHQ) can effectively inhibit lipid oxidation [5]. However, these synthetic antioxidants are restricted by legislative rules because of the doubts over their toxicity and carcinogenicity in many countries [6,7]. Thus, in order to protect human beings against oxidative damage, considerable attention has been paid to explore the natural and safer antioxidants. Among the dietary antioxidants, phenolic compounds are the most abundant natural antioxidants [8].

Phenolic compounds naturally occur in all plant material, and are prominently ubiquitous in fruits, vegetables, seeds, and herbs, but also in plant products, such as beverages, wine, cocoa [9]. These compounds are potent antioxidants and play an important role in human nutrition as preventative agents against several diseases, protecting the body tissues against oxidative stress [10]. As an important group of secondary metabolites presenting in *Lilium*, phenolic compounds play an important role in the quality and nutrition value of lily species [11].

Lily, a perennial ornamental crop belonging to the Liliaceae, has great ornamental, medicinal and edible value. The genus *Lilium*, which includes approximately 100 species, is native to Asia, Europe, and North America in the Northern Hemisphere. China with about 55 species is the diversity centre of wild *Lilium* in the World [12]. Lily bulbs, have been extensively used as both a food and a traditional Chinese medicine for many centuries in China, due to their health promoting properties to treat bronchitis, pneumonia and provide nourishment [13,14]. Recent reports additionally disclose antibacterial and anti-inflammatory activities of the methanol extract of lily bulbs [11]. Up to now, alkaloids, steroidal saponins, flavonoids and polysaccharides have been characterized and isolated from *L*. *lancifolium* and *L*. *brownii* var. *colchesteri* [14,15,16]. As an important Chinese wild resource, *Lilium* species are yet to receive sufficient attention. Furthermore, little information about the antioxidant activity of the phenolic compounds obtained from *Lilium* species is available. This is not conducive to the application of these valuable species. Therefore, the development of studies of the antioxidant activity in *Lilium* species is very topical.

The objective of this study was to evaluate the phenolics content and antioxidant potential of six *Lilium* species native to China. Hopefully, this study will provide sufficient experimental evidence of antioxidant activity and potential for further development and utilization of these *Lilium* species. 

## 2. Results and Discussion

### 2.1. Total Phenolic Content (TPC), Total Flavonoid Content (TFOC) and Total Flavanol Content (TFAC)

Methanol is one of the most widely used solvents and was found to be more effective for extracting phenolics linked to polar fibrous matrices [17]. The samples were freeze-dried before extraction, since previous research has shown that freezing samples may lead to higher extraction efficiencies for total phenolics, because of enhanced plant cell rupture during ice crystal formation and melting, with improved solvent access and extraction [18].

Phenolics, such as flavonoids, phenolic acids, and tannins, are considered to be major contributors to the antioxidant capacity [19]. The correlative phenols composition (including TPC, TFOC and TFA) of the extracts from different *Lilium* samples were examined and shown in Table 1.

The TPC of all samples tested in this study ranged from 2,017.17 to 10,381.49 mg gallic acid equivalent (GAE)/100 g dw. The *L. regale* had the highest amount of TPC followed by *L. pumilum*, *L. concolor*, *L. lancifolium* and *L. leucanthum*, whereas *L. davidii var. unicolor* had the lowest TPC. Compared with other species of the same family (Liliaceae) with lily, the TPC measured in the lily bulbs was greater than that in the onion and shallot, which were reported recently [20]. 

Similar to the TPC, the TFOC of *L. regale* was significantly higher than that in the other tested *Lilium* species, with the mean of 1,428.21 mg rutin equivalent (RE)/100 g dw. The TFOC of the other five species were only 413.45, 521.19, 150.53, 227.24 and 339.13 mg RE/100 g dw for *L. concolor*, *L. leucanthum*, *L. davidii var. unicolor*, *L. lancifolium* and *L. pumilum*, respectively. Flavonoids, known for their antioxidant and antiinflammatory activities, are implicated to the maintenance of health. They also can inhibit the Low Density Lipoprotein (LDL), the oxidation and impart the cardio protective effects [21]. Our results indicate that the high concentration of flavonoids in lily bulbs (range from 0.15 to 1.43% of the dry weight) somewhat substantiates its use in natural antioxidant products.

Flavanols, a subgroup of the flavonoid family, have been demonstrated positive effects with human health, including the recovery of endothelial function, improvements in insulin sensitivity, decreased blood pressure, and reductions in platelet aggregation [22]. The content of total flavanols was estimated by vanillin-HCl assay and described as TFAC (Table 1). The TFAC of all lily bulb extracts varied from 66.07 mg (+)-catechin equivalent (CE)/100 g dw to 407.25 mg CE/100 g dw and the order of TFAC decreased as *L. regale* > *L. concolor* > *L. pumilum* > *L. lancifolium* > *L. leucanthum* > *L. davidii var. unicolor*.

### 2.2. Antioxidant Activities of Lily Bulb Extracts 

Biological systems contain a multiplicity of antioxidant systems that may be involved in complex interactions, including synergistic or antagonistic reactions in the food matrix [23]. Different antioxidant activities were analyzed by different methods which have different reaction characteristics and mechanisms, and there is no universal assay that could accurately reflect all the antioxidants in a complex system. Therefore, it is necessary to use at least two complementary methods to evaluate the antioxidant capacity *in vitro*. In this study, four antioxidant assays, such as DPPH free radical scavenging activity, ABTS radical cation scavenging activity, cupric-reducing antioxidant capacity (CUPRAC) and hydroxyl radical scavenging activity (HRSA) were applied to accurately evaluate the antioxidant properties of six *Lilium* species.

#### 2.2.1. DPPH Radical-Scavenging Activity

The effect of antioxidants on DPPH radical scavenging is generally due to their hydrogen-donating ability. High value of Trolox equivalent antioxidant capacity (TEAC) indicates that the antioxidants acting as a hydrogen donor could terminate the oxidation process by converting free radicals to their stable forms [24]. In this study, all lily bulb extracts, at the testing concentration, were capable of directly reacting with and quenching DPPH^•^. The antioxidant activity of all extracts determined as DPPH radical scavenging ability ranged from 404.48 to 600.33 μmol trolox equivalent (TE)/100 g dw (Table 2). The highest antioxidant activity was found for the bulb extract of *L. regale*, followed by *L. pumilum*, *L. lancifolium*, *L. leucanthum*, and *L. concolor*, while *L. davidii var. unicolor* yielded the lowest antioxidant capacity.

#### 2.2.2. ABTS Radical-Scavenging Activity 

ABTS radical cation scavenging assay is an excellent tool for determining the antioxidant activity of hydrogen-donating antioxidants and of chain breaking antioxidants [25]. All tested *Lilium* species had significant radical cation scavenging activities, and individual species might significantly differ in their ABTS^•+^ scavenging capacities (Table 2). The greatest ABTS^•+^ scavenging capacity of 1,173.28 μmol TE/100 g dw was detected in a *L. regale* sample, while the *L. davidii var. unicolor* sample had the lowest ABTS^•+^ scavenging capacity of 848.49 μmol TE/100 g dw. Based on the mean value of each extract sample, the rank of radical cation scavenging activity was *L. regale* > *L. concolor* > *L. pumilum* > *L. lancifolium* > *L. leucanthum* > *L. davidii var. unicolor*. The radical scavenging of all species on ABTS^•+^ in Table 2 showed a different order with the results of the DPPH^•^ assay. *L. concolor* had a higher antioxidant capacity than other species except *L. regale*. These differences could be attributed to the different stoichiometry reactions between the lily bulb extracts and the DPPH^•^ and ABTS^•+^. In addition, the compositional differences in extracts and their solubility in different testing systems may also affect their capacities to act as antioxidants [26,27].

#### 2.2.3. Cupric ion Reducing Antioxidant Capacity (CUPRAC)

The reducing power property indicates that the antioxidant compounds are electron donors and can reduce the oxidized intermediates of the lipid peroxidation process. These methods focus on detecting the reducing ability of the antioxidant, mainly the reducing ability of iron and copper ions. Currently, FRAP assay is the most widely used method for determining the reducing power of antioxidants. However, the FRAP method has two major flaws: (1) the assay is conducted at acidic pH (3.6) to maintain iron solubility; (2) FRAP assay does not measure thiol antioxidants, such as glutathione [28]. Compared to the ferric reducing antioxidant potency (FRAP) assays based on the ferric-ferrous system, the method of detecting antioxidant capacity from the reducing ability for copperions is free of the shortcomings. We found that the cupric-reducing potential of the bulb extracts was similar to the ABTS-scavenging activity except *L. pumilum* and *L. concolor*, which showed an opposite order in these two assays. In the cupric-reducing potential assay, significant differences were observed among the six *Lilium* species. The rank order based on the average TE values was as follows: *L. regale* > *L. pumilum* > *L. concolor* > *L. lancifolium* > *L. leucanthum* > *L. davidii var. unicolor*. *L. regale* possessed the strongest (1,438.01 μmol TE/100 g dw) reducing activity, while *L. davidii var. unicolor* had the weakest (only 595.61 μmol TE/100 g dw). Several researchers confirmed that CUPRAC is associated with gallic and chlorogenic acid content. Therefore, these phenolic acids would make a certain contribution in the process of cupric reduction [29].

#### 2.2.4. Hydroxyl Radical Scavenging Activities (HRSA)

The hydroxyl radical is an extremely reactive free radicals formed in biological systems and has been implicated as one highly damaging species in free radical pathology, capable of damaging almost every molecule found in living cells [30]. Detection of the hydroxyl radical was via a salicylate probe generated from a reaction between an iron(II)-EDTA complex and H_2_O_2_. The resultant hydroxyl radicals attack both the salicylate probe and the hydroxyl radical scavengers that are incubated in the solution. Radical scavengers compete with salicylate for the hydroxyl radical produced [31]. The hydroxyl radical scavenging activities of all bulb extracts are shown in Table 2. There were significant difference amongst the samples (*p* < 0.05). The highest hydroxyl radical scavenging activity in *L. regale* (53.22 ± 0.99%) was 2.4-fold higher than that in *L. davidii var. unicolor* (22.45 ± 0.60%). The hydroxyl radical scavenging of bulb extracts from the six species decreased as: *L. regale* > *L. concolor* > *L. pumilum* > *L. leucanthum* > *L. lancifolium* > *L. davidii var. unicolor*. 

It is now well accepted that in plants, phenolics constitute the main bioactive phytochemicals that have been proven effective in the prevention of certain chronic diseases such as coronary heart diseases, cancers and diabetes, because of their free radical-scavenging activities [18]. The results of this study showed that the methanol bulb extracts of six *Lilium* species had strong antioxidant activity, including DPPH radical, ABTS radical cation scavenging, hydroxyl radical scavenging activities and cupricion reducing capacity. Among the tested *Lilium* species, *L. regale* had the greatest antioxidant activities, while *L. davidii var. unicolor* owned the lowest antioxidant capacities. Compared with some commonly consumed vegetables, the six tested *Lilium* species had the higher antioxidant capacities than potato and carrot, but lower than some other vegetables, such as tomato, green bean, spinach, kale, broccoli and rhubarb, by measuring their radical cation scavenging activity [32]. Lily bulbs have been used as a vegetable for several centuries in local China, thus the results in this study indicate that lily bulb could be a potential source of natural antioxidant foods and also provide data to health professionals and food policy makers in China for encouraging the population to consume edible lily bulbs as well as, promoting the preservation of such *Lilium* species.

### 2.3. Identification and Determination of Phenolic Constituents in Extracts

**Figure 1:**
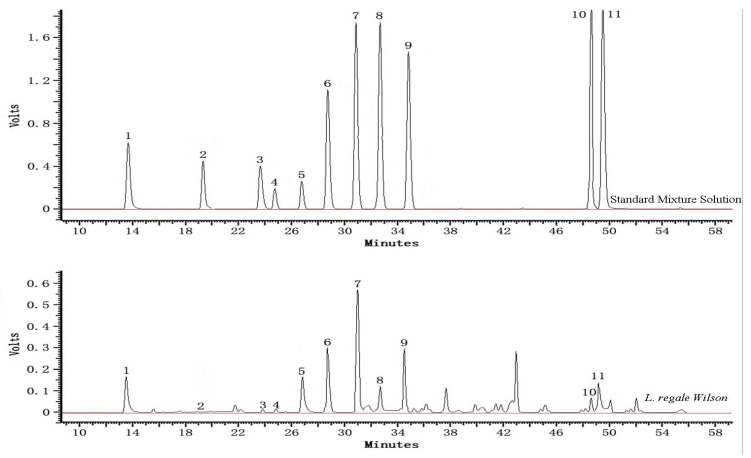
HPLC trace of individual polyphenolic constituents (1, gallic acid; 2, rutinoside; 3, (+)-catechin; 4, chlorogenic; 5, (−)-epicatechin; 6, myricetin; 7, rutin; 8, *p*-coumaric acid; 9, quercetin; 10, phloridzin; 11, kaempferol) of the standard mixture solution and *L. regale* Wilson.

To further understand the phenolics contained in the bulbs of *Lilium* species from rural China, all the extracts were analyzed by HPLC. Three phenolic acid (gallic acid, *p*-coumaric acid and chorogenic acid), five flavonols (rutinoside, myricetin, rutin, quercetin, and kaempferol), two monomeric flavanols [(+)-catechin and (−)-epicatechin] and one chalcone (phloridzin) were quantified. The representative chromatograms of the standard mixture solution and *L. regale* extract separation are depicted in Figure 1 and the phenolic contents of *Lilium* species are listed in Table 3. Statistically significant differences were found between the species analyzed for each compound assayed.

In general, rutin and kaempferol were the most predominant phenolic compounds found in lily bulb extracts, ranging from 0.96 to 20.98 mg/100 g dw and 1.30—12.48 mg/100 g dw, respectively. Rutin in *L. regale* was the maximum among the six *Lilium* species, reaching values five times higher than the amounts detected in the other species analyzed. Rutin is a type of flavonol with various biological activities that may protect against spatial memory impairment accompanying hippocampal pyramidal neuron loss [33]. Kaempferol was mainly abundant in *L. lancifolium* and *L. pumilum* (8.03 and 12.48 mg/100 g dw, respectively). The levels of quercetin in all samples were low, ranging from 0.89 to 6.20 mg/100 g dw. Kaempferol and quercetin flavonols have been reported to effectively recycle vitamin E (an antioxidant) and are also known to reduce inflammation, tumorogenesis and cell damage caused by oxidation [34,35]. These compounds have been found in Easter lily flowers and our results were consistent with the findings of previously published study [36]. *L. regale* contained the highest concentration of myricetin (6.42 mg/100 g dw), followed by *L. davidii var. unicolor*, *L. pumilum*, *L. leucanthum* and *L. concolor*, *L. lancifolium* yieded the lowest content, with the value of 0.81 mg/100 g dw. Minor quantities of rutinoside (0.82–1.09 mg/100 g dw) were found in some of the bulb extracts, these compounds not being present in all *Lilium* species. Phloridzin was detected and quantified in all bulb extracts except *L. pumilum*. *L. regale* had the highest concentration, with the value of 4.45 mg/100 g dw, reaching five times higher than *L. davidii var. unicolor*, which gained the lowest content (0.88 mg/100 g dw). The study demonstrates that phloridzin has the high activity in the oxygen radical absorbance capacity (ORAC) assay and DPPH free radical scavenging activity [37].

(+)-Catechin and (−)-epicatechin were the typical monomeric flavanol compounds identified in the bulbs of the *Lilium* species analyzed. (+)-Catechin and (−)-epicatechin have a positive correlation with DPPH and ABTS^+^ scavenging capacity and reducing power and are noted for their high antibacterial activity against *Escherichia coli*, *Pseudomonas aeruginosa* and *Staphylococcus aureus* [33]. (+)-Catechin was the most abundant monomeric flavanol compound identified in *L. regale* (1.26 mg/100 g dw), followed by *L. lancifolium* and *L. concolor* (with equal value), *L. pumilum* and *L. leucanthum*, whereas the value of *L. davidii var. unicolor* was only 0.82 mg/100 g dw. There were significant difference amongst (−)-epicatechin of the tested samples (*p* < 0.05), with the values ranging from 0.82 to 3.71 mg/100 g dw. Among the six *Lilium* species, *L. concolor* had the highest concentration of (−)-epicatechin, more than four fold than that of *L. davidii var. unicolor*, which contained the lowest amount. 

Regarding the phenolic acids, gallic acid, *p*-coumaric acid and chlorogenic acid were detected and quantified in all the bulb extracts analyzed, except *L. lancifolium* and *L. pumilum*, in which chlorogenic acid were not detected. Natural phenolic acids are strong antioxidants and exhibit potential antifungal, antibacterial, anti-inflammatory and anti-cancer activity [38,39,40]. *p*-Coumaric acid was the most predominant phenolic compound in the tested phenolic acids, ranging from 0.80 to 4.51 mg/100 g dw. *L. regale* had the lowest amount of *p*-coumaric acid, less than one fifth of that in *L. lancifolium*. For chlorogenic acid, *L. concolor* contained the highest concentration, with the value of 2.96 mg/100 g dw, followed by *L. leucanthum* (2.57 mg/100 g dw) and *L. davidii var. unicolor* (1.14 mg/100 g dw), while *L. regale* had the lowest concentration (0.95 mg/100 g dw). The amounts of gallic acid in tested bulb extracts, with mean values (n = 6) of 0.95 mg/100g dw, was approximately 2.2-fold lower than that of *p*-coumaric acid. 

Most of the phenolic compounds herein reported have been shown to possess promising biological properties, especially for their strong antioxidant and antiradical activities *in vitro* and *in vivo* [41,42]. The difference of phenolic composition might explain the different antioxidant abilities of lily bulb extracts observed above. It can be also speculated that phenolic compounds present in the extracts may exert their antioxidant capacity individually as well as synergistically.

### 2.4. Relationships amongst Different Antioxidant Variables

Correlation analysis was used to explore the relationships amongst the different antioxidant variables measured for all bulb extracts of six *Lilium* species (Table 4). CUPRAC and HRSA were significantly correlated with TPC, TFOC and TFAC. The linear correlation coefficients between CUPRAC and TPC, between CUPRAC and TFAC, between HRSA and TFOC, and between HRSA and TFAC were significant at the 0.01 level. ABTS was only significantly correlated with TFAC with the correlation of 0.83 at 0.05 level. However, no significant correlation was found between the components of tested *Lilium* species and DPPH.

Regarding the different methods, the significant correlation between methods was confirmed with four methods (DPPH, ABTS, CUPRAC, and HRSA). The correlation coefficient was 0.77 between DPPH and CUPRAC, and 0.85 between ABTS and CUPRAC at 0.05 level. The correlation between CUPRAC and HRSA was 0.94 at 0.01 level. The correlation coefficients between the phenolic contents and antioxidant capacity among the different methods for quantifying antioxidant capacity seem to be lower than the other reported values [43]. Antioxidant activity is associated not only with the experimental materials but also with the extraction solvents, extract concentration, and reaction time [44]. These factors may contribute to the differences in correlation coefficients between this study and others.

### 2.5. Cluster Analysis

**Figure 2:**
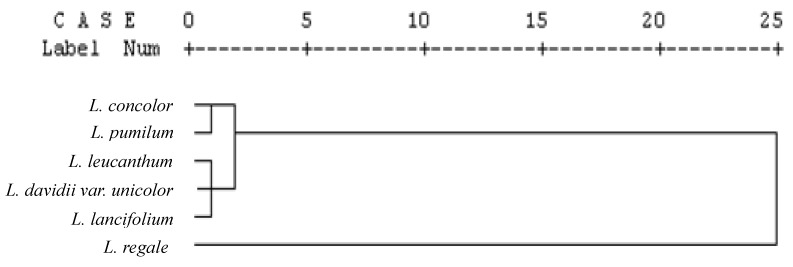
Dendrogram plot visualizing the clustering of the bulb extracts from six *Lilium* Species in this study based on their phenolic composition and antioxidant properties.

Data on phenolic contents and antioxidant capacity were used to carry out a cluster analysis of the *Lilium* species. The dendrogram that was generated by cluster analysis (Figure 2) shows a certain correlation within species belonging to the *Lilium* genus. As the results show, *L. concolor* and *L. pumilum* are arranged in one group characterized by moderate phenolic content and antioxidant capacity. *L. regale* belongs to the group with high phenolic content and strong antioxidant power, while *L. leucanthum*, *L. davidii var. unicolor* and *L. lancifolium* belong to the third group with low phenolic content and weak antioxidant activity.

## 3. Experimental Section 

### 3.1. Plant Materials and Chemicals

**Figure 3:**
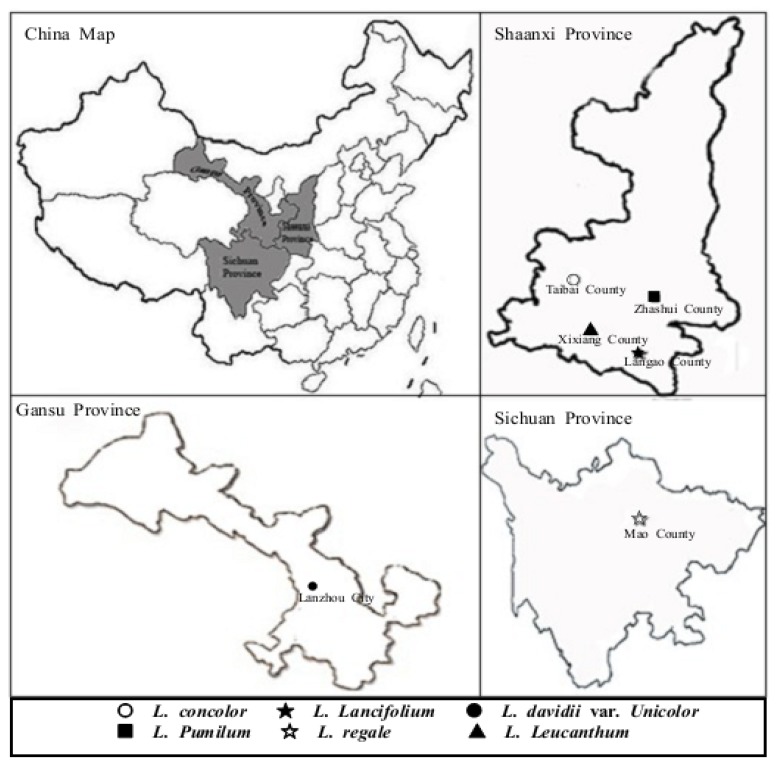
Collection sites of six *Lilium* species native to China.

All bulbs of six *Lilium* species were collected from its original distribution area in Sichuan, Gansu and Shaanxi province on August 2009 (Figure 3), and their taxonomic classification was identified by Professor Lixin Niu at the College of Horticulture, Northwest A&F University. Bulbs of *L. regale* Wilson were collected from Mao County (31° 41′ N, 103° 51′ E), Sichuan and those of *L. davidii* var. *unicolor* were obtained from Lanzhou City (36° 24′ N, 103° 40′ E), Gansu. Other lily bulb samples, including *L. lancifolium* Thunb., *L. leucanthum* (Baker) Baker, *L. concolor* Salisb. and *L. pumilum* DC. were collected from Langao (32° 19′ N, 108° 54′ E), Taibai (34° 02′ N, 107° 19′ E), Xixiang (32° 59′ N, 107° 43′ E) and Zhashui County (33° 41′ N, 109° 06′ E), Shaanxi, respectively. Fresh bulbs of six *Lilium* species were washed with cold water, frozen in liquid nitrogen, freeze-dried (LGJ-10, Songyuan Huasheng Biotechnology Co. Ltd., Beijing, China), grounded into powder using an electrical grinder (JP-250A-8, Jiugong Economy and Trade Co. Ltd., Shanghai, China), stored in labeled plastic bags under vacuum and then stored at −20 °C until extraction.

Sodium carbonate anhydrous, sodium nitrite, aluminium chloride crystalline, sodium acetate trihydrate, chalcanthite, methanol, vanillin and hydrochloric acid were purchased from Tianjin Bodi Chemical Co. (Tianjin, China). Folin-Ciocalteu’s phenol regent, neocuproine, 6-hydroxy-2,5,7,8-tetramethylchromane-2-carboxylic acid (Trolox), 2,2-diphenyl-1-picrylhydrazyl (DPPH), 2,2-azinobis (3-ethyl-benzothiazoline-6-sulfonic acid) (ABTS) and all the phenolic compounds (purity > 97%) were supplied by Sigma-Aldrich (Shanghai, China). All other chemicals were analytical grade supplied by Xi’an Chemical Reagent Co. Ltd. (Xi’an, China).

### 3.2. Preparation of Phenolic Extracts

A fine dried lily bulb powder sample (5 g) was homogenized and extracted with ultrasonic assistance in 50 mL of acidified methanol solution (1 M HCl in 80% methanol) at 25 °C for 1 h in an external water bath. The homogenate was centrifuged at 12,000 rpm for 10 min at 4 °C by using a centrifuge (KDC-140HR, Zhongke Scientific Instruments Co. Ltd., Anhui, China). The residue of extraction was repeated three times under the same conditions. The supernatants of the three runs were combined and analyzed for total phenolics, total flavonoids, total flavanols, and antioxidant activity. 

To determine the individual phenolic compounds by HPLC, 50 mL of the upon extracts was pipetted into a 250 mL evaporation flask and concentrated to a volume of 10 mL on a rotary evaporator (SENCO-R series, Shensheng Biotech Co. Ltd., Shanghai, China) at 40 °C. The enriched extracts (aqueous phase) were then extracted three times with 10 mL of ethyl acetate. Then, the combined organic phases were evaporated to dryness under vacuum. Subsequently, the dried residuals were re-dissolved in 5 mL of methanol (HPLC grade). This methanol solution was filtered through 0.22-µm membranes and analyzed by HPLC for individual phenolic compounds.

### 3.3. Determination of Total Phenolic, Total Flavonoid and Total Flavanols Contents 

Total phenolic content (TPC) was estimated by the Folin–Ciocalteu colorimetric method using gallic acid as standard [45]. In short, distilled water (7.9 mL), *Lilium* bulb extract (0.1 mL), and Folin–Ciocalteu reagent (0.5 mL) were added in a test tube successively. After reaction for 5 min, 20% Na_2_CO_3_ (1.5 mL) was added. The mixture was allowed to react at room temperature in the dark for 2 h, and the absorbance was measured using a UV-visible spectrophotometer (UV-1700, Shimadzu Corp, Kyoto, Japan) at 765 nm against a blank (methanol) similarly prepared. The results were expressed as the equivalent to milligrams of gallic acid per 100 gram of dry weight (mg GAE/ 100 g). 

Total flavonoid content (TFOC) was determined according to the aluminum chloride (AlCl_3_) colorimetric method using rutin as standard [46]. Briefly, in a centrifuge tube, an aliquot of sample solution (1 mL) was mixed with methanol solution (4 mL), NaNO_2_ (0.5 M, 0.3 mL), and AlCl_3_ (0.3 M, 0.3 mL) in sequence. After 5 min, (1 M NaOH, 4 mL) was added to the reaction system, and the absorbance was measured against blank at 510 nm. The results were expressed as the equivalent to milligrams of rutin per 100 gram of dry weight (mg RE/100 g). 

The determination of total flavanol content (TFAC) was performed colorimetrically by the vanillin method using (+)-catechin as a standard [47]. In this procedure the vanillin reagent (5.0 mL, 0.5 g of reagent and 200 mL of 4% HCl methanol) was added to methanolic extract (1 mL) and mixed well. Similarly, a blank was prepared by adding 4% HCl in methanol (5 mL) to methanolic *Lilium* bulb extracts (1 mL). The sample and blank absorbances were read at 500 nm after 20 min in the dark at room temperature. The absorbance of the blank was subtracted from that of the sample and the results were expressed as the equivalent to milligrams of (+)-catechin per 100 gram of dry weight (mg CE/100 g).

### 3.4. Antioxidant Activity Evaluation

DPPH^·^ scavenging activity was analyzed according to the slightly modified method of Brandwilliams, Cuvelier, and Berset [48]. Briefly, an aliquot of sample (0.1 mL) was added to a 6.25 × 10^−5^ M solution of DPPH^·^ in methanol (3.9 mL). A control sample containing the equal volume of methanol in place of sample was used to measure the maximum DPPH^·^ absorbance. After a 30 min reaction in the dark, the absorbance at 517 nm was recorded to determine the concentration of remaining DPPH^·^. The antioxidant capacity was expressed as micromoles trolox equivalents (TE)/100 gram sample of dry weight.

ABTS free radical scavenging activity was carried out using the minor modified method of Re *et al.* [49]. ABTS radical cation (ABTS^·+^) was produced by reacting 7 mM ABTS solution (5 mL) with 140 mM potassium persulfate aqueous solution (88 µL) and allowing the mixture to stand in the dark at room temperature for 12 h before use. The ABTS^·+^ solution was diluted with methanol to give an absorbance at 734 nm of 0.70 ± 0.02 in a 1-cm cuvette at 732 nm. After addition of 0.1 mL of sample to 3.9 mL of diluted ABTS**^·^**^+^ solution, the absorbance was measured at exactly 6 min. Results were expressed as micromoles trolox equivalents (TE)/100 gram sample of dry weight.

The cupricion reducing capacity was determined as described by Apak, Guculu, Ozyurek, and Karademir [50], with slight changes. Briefly, to a test tube, 5 mM CuSO_4_, 3.75 mM neocuproine, NH_4_Ac buffer (1 M, pH 7.0) solutions (1 mL each) and distilled water (0.6 mL) were added. About 0.1 mL of sample was added to the initial mixture so as to make the final volume 4.1 mL. After 30 min, absorbance was measure d at 450 nm. Results were expressed in micromoles Trolox equivalents (TE)/100 gram of extract.

HRSA was estimated using the method described by Sroka and Cisowski with a slight modification [51]. Briefly, FeSO_4_ (9 mM, 1 mL), H_2_O_2_ (45 µL, 0.15%), and salicylic acid (9 mM, 1 mL) were sequentially mixed with distilled water (4 mL). Then, *Lilium* bulb extract (1 mL) was added to this mixture and reacted for 30 min at 37 °C. The absorbance of the coloured product was measured at 536 nm, and the percentage of free radical scavenging activity was calculated as follows:

Scavenging effect (%) = [1 − (A_Sample_ − A_Control_)] × 100%

### 3.5. HPLC Assay for Significant Individual Phenolic Compounds

Identification and quantification of phenolic compounds was carried out using a Shimadzu liquid chromatography system (LC-2010AHT, Shimadzu Corp., Kyoto, Japan) equipped with a quaternary pump, a vacuum degasser, an autosampler, a photodiode array detector, a tunable UV-visible detector, and a Hibar RT Lichrospher SB-C18 column (250 mm × 4.0 mm, 5 µm). The standards, gallic acid, (+)-catechin, (−)-epicatechin, quercetin, chlorogenic acid, rutin, rutinoside, *p*-coumaric acid, phloridzin, kaempferol, and myricetin, were dissolved in methanol at a stock concentration of 1 mg/mL. Calibration standard mixture was prepared by appropriate dilutions with methanol from the stock solution.

A gradient solvent system was employed with solvent A being water-acetic acid (99:1, v/v) and solvent B being acetonitrile. The gradient elution conditions were as follows: 0–40 min, 5%–40% B; 40–45 min, 40%–100% B; 45–60 min, 100% B. The wavelength-switching program was employed. The column was held at 40 °C and was flushed at a flow rate of 0.5 mL/min. A volume of 10 µL was injected for each run in triplicate. The PDA detector scanned from 200 to 400 nm. Phenolic compounds were identified by comparing their retention times with those of pure standards and quantification was made by using the external standard method. Calibration was performed by injecting the standards three times at five different concentrations. Results were acquired, processed by the Shimadzu Workstation CLASS-VP 6.12 software and expressed as mg/100 g sample of dry weight.

### 3.6. Statistical Analysis

All analyses were run in triplicate and the results expressed as mean ± standard deviation (SD), were analyzed using SPSS version 16.0 for Windows. One-way analysis of variance (ANOVA) and Duncan’s multiple range tests were used to determine the significance of the difference among samples, with a significance level of 0.05. A two-tailed Pearson’s correlation test was processed to determine the correlations among means. Hierarchical cluster analysis was used to group *Lilium* species.

## 4. Conclusions 

The antioxidant activities and total and individual phenolic contents of bulb extracts from six *Lilium* species were studied. Significant difference exists in the antioxidant capacity as well as in the total phenolic content of bulb extracts of *Lilium* assayed via the DPPH, ABTS, CUPRAC and HRSA methods. *L. regale* had the highest phenolic contents (TPC, TFOC, and TFAC) and the strongest antioxidant capacity, while *L. davidii var. unicolor* had the lowest phenolic contents and the weakest antioxidant activity among the tested species. *L. concolor* and *L. pumilum* were arranged in one group characterized by moderate phenolic content and antioxidant capacity. *L. leucanthum* and *L. lancifolium*, which belonged to be the same group as *L. davidii var. unicolor*, had low phenolic content and weak antioxidant activity. According to the linear correlation analysis, the total flavanol content is the major contributor to the total antioxidant capacity in *Lilium* bulb except for DPPH. Regarding individual phenolic compounds, *L. regale* contained the highest (+)-catechin, myricetin, rutin, quercetin, and phloridzin contents, and those of kaempferol and rutinoside were the highest in the bulb extract of *L. pumilum*, *L. concolor* had the highest chlorogenic acid, and (−)-epicatechin contents, while *L. lancifolium* extract had the highest concentration of *p*-coumaric and gallic acid. HPLC analysis revealed that rutin and kaempferol as the major phenolic components in the extracts. Overall, lily bulbs are good candidates for further development as nutraceutical supplements or antioxidant remedies. Future studies should focus on the assessments of economic benefits and *in vivo* activities of these extracts before their commercial exploitation.

## Figures and Tables

**Table 1:** Contents of total phenolics, flavonoids and flavanols in bulb extracts from six *Lilium* species.

Species	TPC (GAE mg/100 g)	TFOC (RE mg/100 g)	TFAC (CE mg/100 g)
*L. concolor*	3897.60 ± 42.54 c	413.45 ± 2.03 c	296.13 ± 18.17 b
*L. leucanthum*	2336.00 ± 29.28 e	521.19 ± 17.77 b	107.36 ± 8.40 d
*L. davidii var. unicolor*	2017.17 ± 140.20 f	150.53 ± 3.66 f	66.07 ± 9.07 e
*L. regale*	10381.49 ± 49.12 a	1428.21 ± 38.52 a	407.25 ± 10.91 a
*L. lancifolium*	2827.25 ± 55.50 d	227.24 ± 3.66 e	112.12 ± 12.96 d
*L. pumilum*	4177.39 ± 57.19 b	339.13 ± 9.17 d	193.92 ± 7.95 c

GAE mg/100 g, RE mg/100 g and CE mg/100 g represent milligrams of gallic acid equivalents, milligrams of rutin equivalent and milligrams of (+)-catechin equivalent per 100 grams of dry lily bulbs, respectively. Values are means of three replicates ± SD. Different letters (a–f) within the same column indicate significant difference at *p* < 0.05 by Duncan’s test.

**Table 2:** Antioxidant activity determined by the DPPH, ABTS, CUPRAC and HRSA assays of the bulb extracts from six *Lilium* species.

Species	DPPH (TE µmol/100g)	ABTS (TE µmol/100g)	CUPRAC (TE µmol/100g)	HRSA (%)
*L. concolor*	455.31 ± 7.21 d	1143.67 ± 11.28 a	1025.14 ± 45.68 b	40.86 ± 0.52 b
*L. leucanthum*	507.64 ± 6.85 c	889.38 ± 13.42 b	799.34 ± 5.81 d	36.64 ± 0.80 d
*L. davidii var. unicolor*	404.48 ± 14.59 e	848.49 ± 9.17 b	595.61 ± 7.24 e	22.45 ± 0.60 f
*L. regale*	600.33 ± 2.24 a	1173.28 ± 11.41 a	1438.01 ± 16.56 a	53.22 ± 0.99 a
*L. lancifolium*	541.27 ± 3.43 b	1075.51 ± 2.94 a	842.04 ± 8.32 c	26.85 ± 0.79 e
*L. pumilum*	546.51 ± 9.77 b	1091.96 ± 5.70 a	1044.10 ± 11.30 b	37.47 ± 0.82 c

TE µmol/100 g represents micromoles of trolox equivalents per 100 grams of dry bulbs from six *Lilium* species for DPPH and ABTS free radical-scavenging capacity, and cupric-reducing antioxidant capacity (CUPRAC). Hydroxyl radical-scavenging activity (HRSA) was expressed as the percentage of free radical-scavenging activity (%). Values are expressed as means ± SD (n = 3). Means in the same column followed by different letters (a–f) are significantly different (*p* < 0.05).

**Table 3:** Phenolic composition of the bulb extracts from six *Lilium* Species.

Phenolic compounds	Retention time (min)	*L. concolor*	*L. leucanthum*	*L. davidii var. unicolor*	*L. regale*	*L. lancifolium*	*L. pumilum*
Gallic acid	13.57	0.94 ± 0.03 b	0.90 ± 0.02 bc	0.85 ± 0.06 c	0.88 ± 0.06 bc	1.26 ± 0.01 a	0.85 ± 0.06 c
Rutinoside	19.31	0.82 ± 0.05 c	0.97 ± 0.03 b	0.93 ± 0.02 b	1.05 ± 0.07 a	ND	1.09 ± 0.10 a
(+)-catechin	23.73	1.06 ± 0.10 b	0.92 ± .001 c	0.82 ± 0.03 d	1.26 ± 0.05 a	1.06 ± 0.02 b	0.98 ± 0.02 c
Chlorogenic acid	24.48	2.96 ± 0.06 a	2.57 ± 0.19 b	1.14 ± 0.05 c	0.95 ± 0.04 d	ND	ND
(−)-epicatechin	26.84	3.71 ± 0.09 a	1.07 ± 0.03 c	0.82 ± 0.02 e	0.99 ± 0.01 d	1.48 ± 0.03 b	0.86 ± 0.02 e
Myricetin	28.70	0.82 ± 0.02 c	1.02 ± 0.03 c	2.31 ± 0.28 b	6.42 ± 0.35 a	0.81 ± 0.02 c	1.04 ± 0.08 c
Rutin	31.00	3.36 ± 0.30 c	1.35 ± 0.33 e	0.96 ± 0.03 e	20.98 ± 1.00 a	2.36 ± 0.17 d	4.48 ± 0.20 b
*p*-Coumaric acid	32.63	3.91 ± 0.20 b	1.45 ± 0.06 c	1.26 ± 0.20 c	0.80 ± 0.07 a	4.51 ± 0.31 a	0.82 ± 0.02 d
Quercetin	34.61	ND	1.56 ± 0.20 c	0.89 ± 0.02 d	6.20 ± 0.20 a	2.38 ± 0.19 b	0.96 ± 0.03 d
Phloridzin	48.88	1.03 ± 0.10 c	1.02 ± 0.08 c	0.88 ± 0.04 c	4.45 ± 0.28 a	1.92 ± 0.19 b	ND
Kaempferol	49.44	3.20 ± 0.20 d	1.45 ± 0.19 e	1.30 ± 0.20 e	6.86 ± 0.31 c	8.03 ± 0.39 b	12.48 ± 0.90 a

Values, in mg/100 g dw, are expressed as means ± SD (n = 3). Means in the same line followed by different letters (a–e) are significantly different (*p* < 0.05). ND = Not detected.

**Table 4:** Linear correlation coefficients between phenolic composition and antioxidant capacity (panel A), and among the different methods for quantifying antioxidant capacity (panel B).

	DPPH	ABTS	CUPRAC	HRSA
***Panel A***				
TPC	0.70	0.68	0.94 **	0.86 *
TFOC	0.68	0.51	0.87 *	0.89 **
TFAC	0.54	0.83 *	0.95 **	0.92 **
***Panel B***				
DPPH	1.00			
ABTS	0.63	1.00		
CUPRAC	0.77 *	0.85 *	1.00	
HRSA	0.65	0.69	0.94 **	1.00

* Correlation is significant at the 0.05 level. ** Correlation is significant at the 0.01 level.

## References

[1] Bagchi D., Bagchi M., Stohs S.J., Das D.K., Ray S.D., Kuszynski C.A., Joshi S.S., Pruess H.G. (2000). Free radicals and grape seed proanthocyanidin extract: Importance in human health and disease prevention. Toxicology.

[2] Meng J.F., Fang Y.L., Qin M.Y., Zhuang X.F., Zhang Z.W. (2012). Varietal differences among the phenolic profiles and antioxidant properties of four cultivars of spine grape (*Vitis davidii* Foex) in Chongyi County (China). Food Chem..

[3] Devasagayam T.P.A., Tilak J.C., Boloor K.K., Sane K.S., Ghaskadbi S.S., Lele R.D. (2004). Free radicals and antioxidants in human health: Current status and future prospects. JAPI.

[4] Halliwell B., Gutteridge J., Cross C. (1992). Free radicals, antioxidants, andhuman disease: Where are we now?. J. Lab. Clin. Med..

[5] Formanek Z., Kerry J.P., Higgins F.M., Buckley D.J., Morrissey P.A., Farkas J. (2001). Addition of synthetic and natural antioxidants tocopheryl acetate supplemented beef patties: Effects of antioxidants and packaging on lipid oxidation. Meat Sci..

[6] Ito N., Fukushima S., Tsuda H. (1985). Carcinogenicity and modification of the carcinogenic response by BHA, BHT and other antioxidants. Crit. Rev. Toxicol..

[7] Hao P.P., Ni J.R., Sun W.L., Huang W. (2007). Determination of tertiary butylhydroquinone in edible vegetable oil by liquid chromatography/ion trap mass spectrometry. Food Chem..

[8] Fiorentino A., D’Abrosca B., Pacifico S., Mastellone C., Piscopo V., Caputo R., Monaco P. (2008). Isolation and structure elucidation of antioxidant polyphenols from quince (*Cydonia vulgaris*) Peels. J. Agric. Food Chem..

[9] Bravo L. (1998). Polyphenols: Chemistry, dietary sources, metabolism, and nutritional significance. Nutr. Rev..

[10] Ares G., Barreiro C., Deliza R., Gámbaro A. (2009). Alternatives to reduce the bitterness, astringency and characteristic flavour of antioxidant extracts. Food. Res. Int..

[11] Luo J.G., Li L., Kong L.Y. (2012). Preparative separation of phenylpropenoid glycerides from the bulbs of *Lilium lancifolium* by high-speed counter-current chromatography and evaluation of their antioxidant activities. Food Chem..

[12] Rong L.P., Lei J.J., Wang C. (2011). Collection and evaluation of the genus *Lilium* resources in Northeast China. Genet. Resour. Crop Evol..

[13] Chau C.F., Wu S.H. (2006). The development of regulations of Chinese herbal medicines for both medicinal and food uses. Trends Food Sci. Technol..

[14] You X., Xie C., Liu K., Gu Z. (2010). Isolation of non-starch polysaccharides from bulb of tiger lily (*Lilium lancifolium* Thunb) with fermentation of Saccharomyces cerevisiae. Carbohyd. Polym..

[15] Mimaki Y., Sashida Y. (1991). Steroidal saponins and alkaloids from the bulbs of *Lilium brownii* var. *colchesteri*. Chem. Pharm. Bull..

[16] Niu L.X., Li Z.N., Li H.J., Zhang Y.L. (2007). Study on ultrasonic wave extraction of flavonoids from the bulb of *Lilium lancifolium*. Zhongyaocai.

[17] Hussein L., Fattah M., Salem E. (1990). Characterization of pure anthocyanidins isolated from the hulls of faba beans. J. Agric. Food Chem..

[18] Asami D.K., Hong Y.J., Barret D.M., Mitchell A.E. (2003). Comparison of the total phenolic and ascorbic acid content of freeze-dried and air-dried marionberry, strawberry, and corn grown using conventional, organic, and sustainable agricultural practices. J. Agric. Food Chem..

[19] Velioglu Y.S., Mazza G., Gao L., Oomah B.D. (1998). Antioxidant activity and total phenolics in selected fruits, vegetables, and grain products. J. Agric. Food Chem..

[20] Lu X.N., Wang J., Al-Qadiri H.M., Hamzah M.A.Q., Ross C.F., Powers J.R., Tang J.M., Rasco B.A. (2011). Determination of total phenolic content and antioxidant capacity of onion (*Allium cepa*) and shallot (*Allium oschaninii*) using infrared spectroscopy. Food Chem..

[21] Kondo K., Hirano R., Matsumoto A., Igarashi O., Itakura H. (1996). Inhibition of LDL oxidation by cocoa. Lancet.

[22] Christian H., Carl L.K., Malte K. (2010). Flavanols and cardiovascular disease prevention. Eur. Heart J..

[23] Lutz M., Jorquera K., Cancino B., Ruby R., Henriquez C. (2011). Phenolics and antioxidant capacity of table grape (*Vitis vinifera* L.) cultivars grown in Chile. J. Food Sci..

[24] Li H., Wang X.Y., Li Y., Li P.H., Wang H. (2009). Polyphenolic compounds and antioxidant properties of selected China wines. Food Chem..

[25] Leong L.P., Shui G. (2002). An investigation of antioxidant capacity of fruits in Singapore markets. Food Chem..

[26] Lissi E.A., Modak B., Torres R., Escobar J., Urzua A. (1999). Total antioxidant potential of resinous exudates from *Heliotropium* sp. A comparison of ABTS and DPPH methods. Free Radic. Res..

[27] Mathew S., Abraham T.E. (2006). *In vitro* antioxidant activity and scavenging effects of *Cinnamomum verum* leaf extract assayed by different methodologies. Food Chem. Toxicol..

[28] Prior R.L., Wu X.L., Schaich K. (2005). Standardized methods for the determination of antioxidant capacity and phenolics in foods and dietary supplements. J. Agric. Food. Chem..

[29] Mustafa O., Burcu B., Cubilay G., Resat A. (2008). Hydroxyl radical scavenging assay of phenolics and flavonoids with a modifiedcupric reducing antioxidant capacity (CUPRAC) method using catalase for hydrogen peroxide degradation. Anal. Chim. Acta.

[30] Tedesco I., Russo M., Russo P., Iacomino G., Russo G.L., Carraturo A., Faruolo C., Moio L., Palumbo R. (2000). Antioxidant effect of red wine polyphenols on red blood cells. J. Nutr. Biochem..

[31] Mustafa O., Burcu B., Cubilay G., Resat A. (2008). Hydroxyl radical scavenging assay of phenolics and flavonoids with a modified cupric reducing antioxidant capacity (CUPRAC) method using catalase for hydrogen peroxide degradation. Anal. Chim. Acta.

[32] Zhou K.Q., Yu L.L. (2006). Total phenolic contents and antioxidant properties of commonly consumed vegetables grown in Colorado. LWT-Food Sci. Technol..

[33] Ksouri R., Falleh H., Megdiche W., Trabelsi N., Mhamdi B., Chaieb K. (2009). Antioxidant and antimicrobial activities of the edible medicinal halophyte *Tamarix gallica* L. and related polyphenolic constituents. Food Chem. Toxicol..

[34] Javanovic S.V., Steenken S., Hara Y., Simic M.G. (1996). Reduction potential of flavonoid and model phenoxyl radicals. Which ring in Flavonoids is responsible for antioxidant activity?. J. Chem. Soc. Perk. T..

[35] Dempke W., Rie C., Grothey A., Schmoll H.J. (2001). Cyclooxygenase-2: A Novel target for cancer chemotherapy?. J. Cancer Res. Clin..

[36] Francis J.A., Rumbeiha W., Nair M.G. (2004). Constituents in Easter lily flowers with medicinal activity. Life Sci..

[37] Bernonville T.D., Guyot S., Paulin J.P., Gaucher M., Loufrani L., Henrion D., Derbré S., Guilet D., Richomme P., Dat J.F. (2010). Dihydrochalcones: Implication in resistance to oxidative stress and bioactivities against advanced glycation end-products and vasoconstriction. Phytochemistry.

[38] Cao X.F., Wang Y.S., Li S.W., Chen C.S., Ke S.Y. (2011). Synthesis and Biological Activity of a Series of Novel *N*-Substituted β-Lactams Derived from Natural Gallic Acid. J. Chin. Chem. Soc..

[39] Kadoma Y., Fujisawa S.A. (2008). Comparative Study of the Radical-scavenging activity of the phenolcarboxylic acids caffeic Acid, *p*-coumaric acid, chlorogenic acid and ferulic acid, with or without 2-mercaptoethanol, a thiol, using the induction period method. Molecules.

[40] Gonthier M., Verny M., Besson C., Remesy C., Scalbert A. (2003). Chlorogenic acid bioavailability largely depends on its metabolism by the gut microflora in rats. J. Nutr..

[41] Rice-Evans C.A., Miller N.J., Paganga G. (1997). Antioxidant properties of phenolic compounds. Trends Plant Sci..

[42] Dai J., Mumper R.J. (2010). Plant phenolics: Extraction, analysis and their antioxidant and anticancer properties. Molecules.

[43] Du G.R., Li M.J., Ma F.W., Liang D. (2009). Antioxidant capacity and the relationship with polyphenol and Vitamin C in *Actinidia* fruits. Food Chem..

[44] Jayaprakasha G.K., Singh R.P., Sakariah K.K. (2001). Antioxidant activity of grape seed (*Vitis vinifera*) extracts on peroxidation models *in vitro*. Food Chem..

[45] Singleton V.L., Orthofer R., Lamuela-Raventos R.M. (1999). Analysis of total phenols and other oxidation substrates and antioxidants by means of Folin-Ciocalteu reagent. Method Enzymol..

[46] Chang C., Yang M., Wen H., Chern J. (2002). Estimation of total flavonoid content in propolis by two complementary colorimetric methods. J. Food Drug Anal..

[47] Price M.L., Scoyoc S.V., Butler L.G. (1978). A critical evaluation of the vanillin reaction as an assay for tannin in sorghum grain. J. Agric. Food Chem..

[48] Brand-Williams W., Cuvelier M.E., Berset C. (1995). Use of a free radical method to evaluate antioxidant activity. LWT-Food Sci. Technol..

[49] Re R., Pellegrini N., Proteggente A., Pannala A., Yang M., Rice-Evans C. (1998). Antioxidant activity applying an improved ABTS radical cation decolorization assay. Free Radic. Biol. Med..

[50] Apak R., Guclu K., Ozyurek M., Karademir S.E. (2004). Novel total antioxidant capacity index for dietary polyphenols and vitamins C and E, using their cupricion reducing capability in the presence of neocuproine: CUPRAC method. J. Agric. Food Chem..

[51] Sroka Z., Cisowski W. (2003). Hydrogen peroxide scavenging, antioxidant and anti-radical activity of some phenolic acids. Food Chem. Toxicol..

